# Dosimetric characterization of a liquid‐filled ion chamber array post upgrade and evaluation of improvements for stereotactic plan verification

**DOI:** 10.1002/acm2.70371

**Published:** 2025-12-18

**Authors:** Morgan Healy, Michael Roche, Darren Coen

**Affiliations:** ^1^ CUH‐UCC Cancer Centre Cork University Hospital Wilton Cork Ireland

**Keywords:** Octavius 1000 SRS SABR PSQA

## Abstract

**Background:**

Stereotactic ablative radiotherapy (SABR) is a technique developed for delivery of high doses of radiation to target volumes. Standard delivery methods for SABR include dynamic conformal arc therapy (DCAT) and volumetric modulated arc therapy (VMAT) as they allow for meaningful gains in delivery speed and in some instances sparing of normal tissues compared to conventional 3D planning. However, these techniques require complex treatment planning system (TPS) algorithms, as well as sophisticated irradiation methods. As a result, verification of the planned dose distribution prior to treatment is still standard procedure in most clinical settings. Because of the complex nature of SABR VMAT fields, a full 3D dose matrix is advantageous for plan verification. This 3D dose matrix can be obtained with the OCTAVIUS 4D system, associated software, and 1000^SRS^ array.

**Purpose:**

The aim of this study was to compare the dosimetric characteristics of the OCTAVIUS 1000^SRS^ array pre‐ and post‐upgrade and to evaluate any improvements in the array's performance post‐upgrade.

**Methods:**

The array's performance was tested post‐upgrade and results were compared to pre‐upgrade measurements acquired five years prior at commissioning. This study evaluated the calibration of the central chamber, relative calibration of peripheral chambers, water equivalent depth of the effective point of measurement (EPOM), signal leakage, dose rate linearity, output factors for field sizes ranging from 1.0 × 1.0 to 10.0 × 10.0 cm^2^, and gamma analysis passing rates for ten lung and liver SABR plans.

**Results:**

The EPOM and absorbed dose calibration of the array under reference conditions remain unchanged, but signal leakage for all chambers and relative calibration of off‐axis chambers has improved after the repair. The workflow for VMAT deliveries initially involved multiple calibrations with the appropriate calibration file being selected for each measurement based on the average dose rate of the plan. The array exhibits such improved dose rate linearity post‐upgrade that a single calibration file at 1000 MU/min is now sufficient as the array has a response variation of < ± 0.4% across the range of dose rates expected in clinical plans (700–1300 MU/min). As the dose rate in the measured plane decreases with decreasing field size, other studies have suggested using field size dependent output factor corrections, though our output factor measurements showed agreement < 0.95% with the Monaco treatment planning system for field sizes ≥ 1.5 × 1.5 cm^2^ and we find this is not required. There was also an increase of 9.2% in the gamma analysis passing rates for clinical deliveries using a 2%/1 mm criteria (3D gamma, global dose, and 10% threshold).

**Conclusions:**

The replaced cable and upgraded front foil and detector field have had a positive impact on the dosimetric performance of the 1000^SRS^ array. The unaffected array housing and EPOM means no change is required for positioning and measurement set‐up. The absorbed dose determination under reference conditions has not been impacted but improved dose‐rate linearity, relative calibration of peripheral chambers, and signal leakage mean measurements under non‐reference conditions such as a VMAT delivery are more accurate than before.

## 1 INTRODUCTION

Stereotactic ablative radiotherapy (SABR) is a technique developed for the delivery of high doses of radiation to target volumes. Standard delivery methods for SABR include dynamic conformal arc therapy (DCAT) and volumetric modulated arc therapy (VMAT) as they allow for a more conformal shaping of the dose to the target, with substantial gains in delivery speed and in some instances the sparing of normal tissues compared to conventional 3D planning. However, these techniques require complex treatment planning system (TPS) algorithms, as well as sophisticated irradiation methods. As a result, verification of the planned dose distribution prior to the patient treatment is still standard procedure in most clinical settings.^1^, ^2^, ^3^

At our clinic, SABR patient specific plan verification (PSQA) has been performed using the OCTAVIUS 4D system^4^, ^5^ (PTW‐Freiburg GmbH, Freiburg, Germany). The OCTAVIUS 4D system consists of a 2D detector, the OCTAVIUS 4D rotational phantom, a SRS top for the rotational phantom, a wireless inclinometer, the OCTAVIUS control unit, a DI4000 electrometer (T16039), and the VeriSoft software (v7.2.0.68) which is used to perform the dose reconstruction and the PSQA analysis.^6^ The OCTAVIUS 1000^SRS^ array (T10036) is a 2D detector array with 977 liquid filled ionization chambers. The ionization chambers cover an active area of 11 × 11 cm^2^ with a proposed measurement range of field sizes from 1 × 1 to 10 × 10 cm^2^. The chambers in the central 5.5 × 5.5 cm^2^ region are positioned with a center to center distance of 2.5 mm inline and 3.5 mm diagonally. Outside the central region the chambers are spaced 5.0 mm inline and 7.1 mm diagonally. Each chamber has a size of 2.3 × 2.3 × 0.5 mm^3^ with an effective point of measurement (EPOM) located 9 mm below the surface of the array (10.1 mm water equivalent). The detector has a reported dose‐rate measurement range of 0.1 to 144 Gy/min and a reported dose measurement range from 50 mGy with no upper limit specified for dose delivery. The detector's dose resolution is 0.1 mGy^4^.

A disadvantage with the OCTAVIUS 1000^SRS^ array for SABR plan verification is the non‐linear dose rate response particularly in flattening filter free (FFF) beams routinely used for SABR treatments. The manufacturer specifies non‐linearity of dose rate ≤ ± 0.5% though 3% has been reported in the literature.^7^ This dose rate dependence is due to increased recombination in the liquid filled active volume of the detectors of the array, which is more noticeable across the large range of dose rates utilized in the FFF beams. This can be a disadvantage in SABR plans where the total area, in the measured plane, is in the order of 3 × 3 cm^2^ or in other non‐SABR clinical plans where a large percentage of small segments are delivered. This effect has been identified previously, with a dose rate dependence of 2.8% being reported^7^, ^8^ in a conventional flattening filter beam (CFF). Various solutions have been proposed^9^, ^10^ though implementation is not widespread for practicality reasons. The objective of this study was to characterize the OCTAVIUS 1000^SRS^ detector array and DI400 electrometer after a recent upgrade and to evaluate any improvements for SABR plan verification.

## 2 MATERIALS AND METHODS

The 1000^SRS^ array, serial number 000519, was first purchased in 2019, and commissioned for use with the 6 MV CFF beam (X6 CFF) and 6 MV FFF beam (X6 FFF) on an Elekta Versa HD linear accelerator. The X6 FFF beam has a maximum dose rate of approximately 1300 MU/min. It was returned to the manufacturer for repair in the first quarter of 2024 following a “HV error” message appearing in the VeriSoft software. The manufacturer deemed the detector field to be defective. Corrective maintenance work carried out by PTW involved exchanging the entire detector field and front foil of the 1000^SRS^ array with the latest version. Unfortunately, the manufacturer provided no information as to what the revision number is and what the latest version improved. A schematic diagram showing a cross‐sectional view of the array is provided by Poppe et al.^7^ showing there is a single volume of iso‐octane liquid common to all chambers and the polarizing electrode is an upstream copper foil also common to all chambers. Downstream of the iso‐octane liquid there are 977 copper electrodes, with the electric field between each downstream electrode and the front foil defining the active volume of each chamber. The cable connecting the array to the DI4000 was also replaced. The array was recalibrated in the PTW laboratory before being returned and a new calibration certificate was also provided. The VeriSoft software, inclinometer, and rotational 4D phantom were not affected by the hardware upgrade and are not considered further in this study.

### 2.1 Array calibration

The 1000^SRS^ array comes with a calibration certificate issued by PTW‐Freiburg. During the manufacturer's calibration with a ^60^Co beam and a 10.0 × 10.0 cm^2^ field size, an absolute calibration is conducted on the central chamber of each array and all subsequent chambers in the arrays are then calibrated relative to the central chamber.

#### 2.1.1 Reference calibration

Because of a desire for the array to be traceable to the NPL primary standard,^11^, ^12^, ^13^ a reference calibration on the central chamber of the array was completed.This calibration was completed with the PTW ArrayCal software, according to the procedure outlined in the ArrayCal user manual^14^ with the exception that a 4.0 × 4.0 cm^2^ field size was used instead of the recommended 10.0 × 10.0 cm^2^, as smaller fields are more relevant to SABR treatments. The calibration was carried out for X6 CFF and X6 FFF beams using a Farmer‐type ionization chamber (PTW 30013) and Unidose E electrometer traceable to the NPL primary standard, with the SRS top attached to the rotational phantom. The IPEM/NPL dosimetry protocol was used for determination of absorbed dose,^11^, ^12^, ^13^ the IPEM code of practice includes a correction factor to address difficulties specific to FFF beams.^15^ The SRS top is a solid polystyrene cylinder with a diameter of 8.5 cm, it was assigned a relative electron density (RED) of 1.016 in the TPS. Dose to medium was used with the XVMC photon Monte Carlo algorithm in Monaco (v6.10.01) for 100 MU, 4.0 × 4.0 cm^2^ field size, gantry and collimator at 0°, 2 mm grid size, and 0.2% uncertainty per control point.

#### 2.1.2 Relative calibration

It is possible to create customized in‐house calibration files for the array's relative calibration, which can then be imported into VeriSoft and applied to measurements. To develop these calibration files a dose value is required for each of the 977 chambers within the array. Since it is impractical to achieve a measurement uncertainty equal to or better than the stated uncertainty of the PTW calibration certificate, no relative calibration was completed for the off‐axis chambers in the array. Instead the relative calibration issued by PTW‐Freiburg was considered adequate and verified using a 180° flip test. It should be noted that the calibration certificate issued by PTW in 2024 states that all peripheral chambers are calibrated relative to the central chamber to an uncertainty of ± 0.5% for *k* = 2. This is an improvement over the initial calibration certificate which had an uncertainty of ± 1.0% for *k* = 2 for off‐axis chambers in the 1000^SRS^ array.

For the 180° flip test the array was irradiated with a 15.0 × 15.0 cm^2^ 200 MU field, then rotated 180° clockwise/anti‐clockwise on the treatment couch with its normal axis parallel to the beam while maintaining all other aspects of the set‐up, then another 15.0 × 15.0 cm^2^ 200 MU measurement was delivered. The two measurements were then analyzed in VeriSoft using a gamma analysis, which evaluates each chamber against its corresponding chamber on the contra‐lateral side of the array. The un‐flipped measurement was loaded into dataset B and used as the reference dataset, while the flipped measurement was loaded into dataset A. As each detector element is 2.3 mm wide, a distance‐to‐agreement (DTA) of 0.1 mm was chosen to ensure each chamber is being evaluated against just a single corresponding chamber. A 10% threshold and local dose were chosen for gamma criteria. Gamma results were analyzed for both 0.5% and 1.0% dose‐difference following the calibrations. Measurements were repeated for X6 CFF and X6 FFF at maximum dose rate and X6 FFF at a reduced dose rate.

### 2.2 Water equivalent depth

As the detector field and front foil of the array were replaced with an upgraded model the water equivalent depth of the EPOM was verified. The exterior markings on the array housing were not altered in the repair so the physical depth of the EPOM remains at 9 mm from the top surface, with an area density of 1.12 g/cm^2^ above the detector field^4^ indicating a specified water equivalence of 10.1 mm.

The water equivalent depth of the 1000^SRS^ array was determined pre‐ and post‐upgrade through comparison of percentage depth dose (PDD) data. The array was set‐up at 90.0 cm source‐surface distance (SSD) with ≥ 10.0 cm of backscatter. WTE Bart's Solid Water build up was successively increased from 0 to 50 mm as the array was irradiated with 10.0 × 10.0 cm^2^ fields at 100 MU. The PDD was determined using the central chamber reading. Reference PDDs were taken from water tank scans with a Sun Nuclear SNC125c™ chamber. The water equivalent depth of the array was determined by comparison of the array PDDs to the reference PDDs using a least‐squares fit.

### 2.3 Signal leakage

The array was first switched on, pre‐irradiated with a 10.0 × 10.0 cm^2^ 500 MU field, and zeroed before every measurement. Leakage testing was performed while the detector was stationary for 10 mins and rotated through 360° at the maximum gantry speed to replicate clinical irradiation conditions. Stationary measurements are an average of three measurements, while the rotational measurements are an average of six measurements at maximum gantry speed (three clockwise and three anti‐clockwise). A 360° rotation of the linac gantry takes approximately 1 min to complete.

The manufacturer's specifications for leakage are ≤ 0.5 mGy/min for the central chamber with a maximum of ≤ 2.0 mGy/min for all other chambers, after a zero adjustment.^4^ The manufacturer's specifications were set as the action level and the IPEM 81^16^ guideline of 0.1% was set as the suspension level. For SABR Lung treatments with the X6 FFF beam, dose rates of 10 Gy/min and upwards are expected, creating a suspension level of 10 mGy/min based of the IPEM 81 recommendations.^16^

### 2.4 Dose rate linearity

A dose rate linearity test was performed using the array within solid water, irradiating the detectors with 200 MU at 5.0 cm deep, 95.0 cm SSD, and 10.0 × 10.0 cm^2^ field. The dose rate linearity was evaluated by measuring the central chamber response for multiple dose rates in the X6 FFF beam. The array was replaced with a chamber block and Farmer‐type ionization chamber, and the dose rate linearity test repeated. The manufacture tolerance is specified as ≤ ± 0.5% (following IEC 60731^17^).

The dose per pulse (DPP) was determined using Equation 1, where *D* is the total dose (Gy) for the beam at the level of the detector measured with a Farmer‐type chamber under identical set‐up conditions, *MU* is the number of monitor units delivered, *DR* is the dose rate (MU min^−1^), and *PRF* is the pulse repetition frequency (Hz) acquired with the IC Profiler device (Sun Nuclear Corporation). The purpose of the IC Profiler measurements was solely to determine the PRF for the X6 FFF beam at nominal dose rate before and after the array upgrade.

(1) $$\mathrm{DPP}=\frac{D}{\left(\frac{\mathrm{MU}}{\mathrm{DR}}\right)\times 60\times \mathrm{PRF}}$$

### 2.5 Output factors

The response of the 2D array as a function of field size (S_c,p_) was investigated by delivering 200 MU for square fields ranging from 1.0 × 1.0 to 10.0 × 10.0 cm^2^, normalized to 4.0 × 4.0 cm^2^. Output factors in the SRS top were compared to those calculated using the Monaco XVMC photon Monte Carlo algorithm, whereas output factors measured in solid water were compared to ion chamber measurements. The beam model in the TPS is the Virtual Source Model (v1.6) for a Versa HD linac with Agility multi‐leaf collimator from Elekta's Accelerated Go‐Live product. The calculation parameters were assigned a RED of 1.016 for the phantom material, 0.2% uncertainty per control point, 1 mm grid size for fields ≤ 4.0 × 4.0 cm^2^ or 2 mm grid for fields > 4.0 × 4.0 cm^2^, and a 1.1 mm radius volume of interest (VOI) to extract mean dose values. The IBA CC04 air‐filled ion chamber (0.04 cc) was used for measurements in solid water. Set‐up conditions were 90.0 cm SSD, EPOM at 10.0 cm deep, and > 10.0 cm of backscatter material. Correction factors were applied following the IAEA TRS 483 protocol.^18^

### 2.6 Clinical deliveries

To validate the 1000^SRS^ array under typical clinical conditions nine lung SABR and one liver SABR plans utilizing the X6 FFF beam were evaluated using the gamma analysis with VeriSoft. All clinical deliveries occurred within a 12‐month period; the tens plans were initially measured in the 6‐month period immediately prior to the array upgrade and repeat measurements occurred in the 3‐month period post‐upgrade. There were no changes to the TPS in that period.

Each plan was recalculated on a virtual dataset of the OCTAVIUS phantom with SRS top using the Monaco TPS (Monte Carlo algorithm, 2 mm grid, 3% uncertainty per control point). Before each measurement session began, a daily cross calibration was carried out using a 100 MU, 4.0 × 4.0 cm^2^ field to correct for output variations of linac. This correction was applied using the *k_cross_* factor in Verisoft.

Following delivery, each measurement was compared to the calculated plan with various absolute gamma criteria. The gamma criteria investigated were 2%/1 mm, 2%/2 mm, and 3%/2 mm. A 3D global gamma with a dose threshold of 10% was used in all cases, and no alignment shifts were applied in VeriSoft.

## 3 RESULTS

### 3.1 Array calibration

#### 3.1.1 Reference calibration

Calibration results are presented in Table 1. The 1000^SRS^ array shows agreement within ± 0.75% both before and after the hardware upgrade when compared against either the TPS or the Farmer‐type ion chamber for X6 CFF and X6 FFF.

**TABLE 1:** Results of measurements made with the X6 CFF and X6 FFF beams in the OCTAVIUS rotational phantom with the SRS top compared to an I.C. (thimble ion chamber; PTW 30013) and the TPS (Monaco; phantom assigned RED = 1.016, 2 mm grid size, 0.2% uncertainty per control point). Set‐up conditions were: 4.0 × 4.0 cm^2^ field size, 100 MU, 91.5 cm SSD, and 8.5 cm depth. Uncertainties; *k* = 2.

		Dose (Gy)			Difference	
Energy		I.C.	TPS	1000^SRS^ array	1000^SRS^ vs I.C.	1000^SRS^ vs TPS
X6 CFF	Pre:	0.922 ± 0.018	0.927 ± 0.019	0.921 ± 0.023	0.11%	−0.65%
	Post:	0.920 ± 0.018		0.923 ± 0.023	0.30%	−0.45%
X6 FFF	Pre:	0.947 ± 0.019	0.954 ± 0.019	0.947 ± 0.024	−0.00%	−0.74%
	Post:	0.952 ± 0.019		0.950 ± 0.024	−0.24%	−0.47%

#### 3.1.2 Relative calibration

**FIGURE 1:**
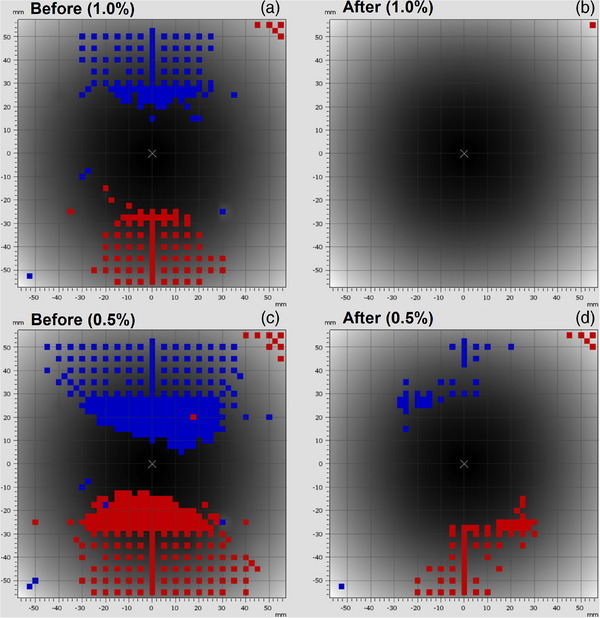
Comparison of the 180° flip test before (a, c at 1270 MU/min) and after (b, d at 1324 MU/min) the upgrade for X6 FFF. Gamma criteria of 0.1 mm DTA, and 1.0 % (a, b) or 0.5% (c, d) local difference are presented. [red = points above local dose difference, blue = points below local dose difference].

**FIGURE 2:**
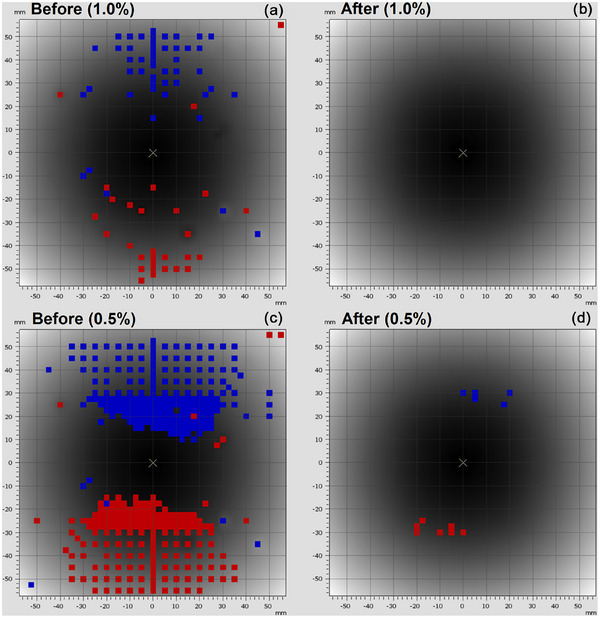
Comparison of the 180° flip test before (a, c at 750 MU/min) and after (b, d at 650 MU/min) upgrade for X6 FFF at reduced dose rate. Gamma criteria of 0.1 mm DTA, and 1.0 % (a, b) or 0.5% (c, d) local difference are presented. [red = points above local dose difference, blue = points below local dose difference].

The results of the 180° flip test to verify the relative calibration before and after the array upgrade are presented for X6 FFF at maximum dose rate in Figure 1 and X6 FFF at half dose rate in Figure 2.

### 3.2 Water equivalent depth

We determined the water equivalent depth of the EPOM pre‐upgrade to be 10.5 ± 0.3 mm (*k* = 2) for both X6 CFF and X6 FFF, and post‐upgrade to be 10.4 ± 0.3 mm for X6 CFF and 10.0 ± 0.3 mm for X6 FFF.

### 3.3 Signal leakage

The array's overall performance was better for stationary measurements compared to rotational measurements, with no difference being observed between clockwise and anti‐clockwise directions. Both pre‐ and post‐upgrade; the central chamber was within action level (0.5 mGy/min) for stationary measurements but between action and suspension level (10 mGy/min) for rotating measurements. At the original commissioning 13 peripheral chambers had readings exceeding suspension level (10 mGy/min) and were deactivated, while no chambers were deactivated post‐upgrade as none exceeded the suspension level. Despite the improvement however, a large number of peripheral chambers pre‐ and post‐upgrade still exceeded the action level (2.0 mGy/min) as shown in Table 2. The peripheral chambers do not appear to have improved post‐upgrade for stationary measurements, but have improved for rotational measurements which are more clinically relevant. The chamber with the maximum reading varied with each measurement. The location of the max reading occurred in both high and low‐resolution areas but was never observed to be the central chamber.

**TABLE 2:** Leakage results for stationary measurements (10 mins duration) and rotating measurements (1 min duration). The action levels for the central and peripheral chamber are 0.5 and 2.0 mGy/min respectively. The suspension level is 10 mGy/min for all chambers.

		Central chamber	Peripheral chambers		
Set‐Up		Reading (mGy/min)	Max reading (mGy/min)	>2.0 mGy/min	>10 mGy/min
Stationary:	Pre:	0.04	7.20	2/976 [0.2%]	0/976 [0.0%]
	Post:	0.00	4.54	98/976 [10%]	0/976 [0.0%]
Rotating:	Pre:	1.49	16.80	168/976 [17%]	13/976 [1.3%]
	Post:	1.13	4.67	48/976 [4.9%]	0/976 [0.0%]

### 3.4 Dose rate linearity

**FIGURE 3:**
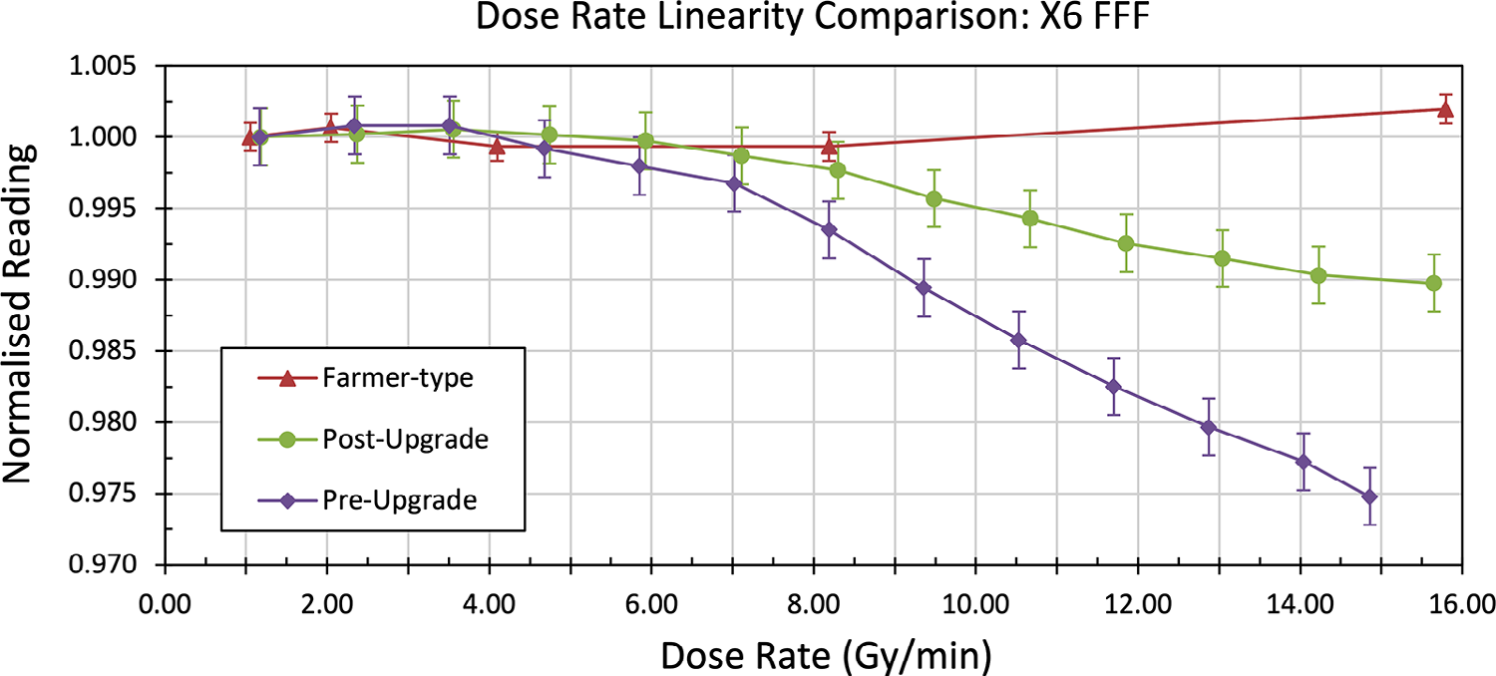
Difference in the dose rate linearity pre‐ and post‐upgrade compared to a thimble ion chamber. All measurements normalised to 100 MU/min. Uncertainties; *k* = 2.

Dose‐rate linearity results are given in Figure 3. The linac PRF was 399.9 Hz for X6 FFF at maximum dose rate, and the DPP was 6.2×10^−4^ Gy/pulse pre‐upgrade and 6.5×10^−4^ Gy/pulse post‐upgrade. The array's dose‐rate non‐linearity improved from 2.52% to 1.02% post‐upgrade, although both measurements are outside the manufacturer's specification of ≤ 0.5%. When the measurement was repeated with a Farmer‐type ion chamber, the dose‐rate linearity was 0.20%.

### 3.5 Output factors

**FIGURE 4:**
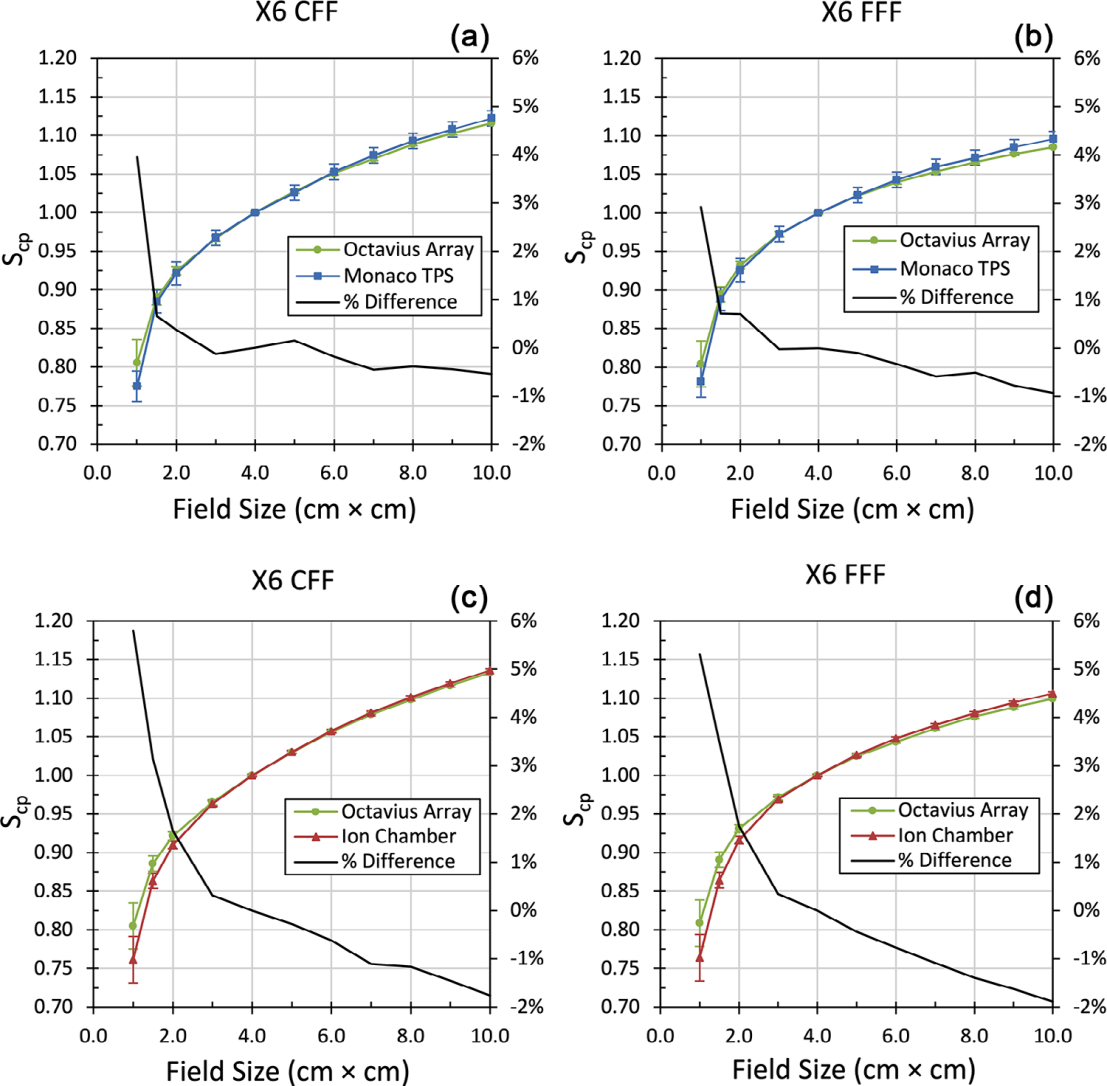
Post‐upgrade output factors (*S*_cp_) compared against the Monaco TPS in the rotational phantom with SRS top (a, b) and against the IBA CC04 ion chamber in solid water at 10.0 cm depth (c, d). Uncertainties; *k* = 2.

The response of the OCTAVIUS 1000^SRS^ array post‐upgrade as a function of field size for both X6 CFF and X6 FFF beam energies are illustrated in Figure 4. The change in response before and after the hardware upgrade is detailed in Table 3. All post‐upgrade output factors show agreement with both the TPS and ion chamber for field sizes > 2.0 × 2.0 cm^2^. As PSQA tests in the clinic compare measurements in the SRS top to the TPS, those output factors are of most relevance, and post‐upgrade the array shows agreement with the Monaco AGL model down to 1.5 × 1.5 cm^2^. Output factors comparing the array pre‐ and post‐upgrade shows maximum differences of –0.92% at 10.0 × 10.0 cm^2^ and –1.19% at 1.0 × 1.0 cm^2^.

**TABLE 3:** Differences in the 1000^SRS^ array output factors (*S*_cp_) pre‐ and post‐upgrade using the rotational phantom with SRS top. All measurements normalised to 4.0 × 4.0 cm^2^. Uncertainties; *k* = 2.

	X6 CFF			X6 FFF		
Field size (cm × cm)	Pre‐	Post‐	Diff.	Pre‐	Post‐	Diff.
1.0	0.805 ± 0.024	0.806 ± 0.024	0.04%	0.814 ± 0.024	0.804 ± 0.024	−1.19%
1.5	0.890 ± 0.009	0.891 ± 0.009	0.12%	0.896 ± 0.009	0.894 ± 0.009	−0.19%
2.0	0.923 ± 0.005	0.925 ± 0.005	0.16%	0.932 ± 0.005	0.932 ± 0.005	0.01%
3.0	0.966 ± 0.003	0.966 ± 0.003	0.08%	0.972 ± 0.003	0.973 ± 0.003	0.07%
4.0	1.000 ± 0.002	1.000 ± 0.002	−	1.000 ± 0.002	1.000 ± 0.002	−
5.0	1.029 ± 0.002	1.027 ± 0.002	−0.19%	1.023 ± 0.002	1.022 ± 0.002	−0.12%
6.0	1.054 ± 0.002	1.051 ± 0.002	−0.33%	1.041 ± 0.002	1.040 ± 0.002	−0.17%
7.0	1.076 ± 0.002	1.070 ± 0.002	−0.61%	1.059 ± 0.002	1.053 ± 0.002	−0.50%
8.0	1.095 ± 0.002	1.089 ± 0.002	−0.56%	1.073 ± 0.002	1.066 ± 0.002	−0.68%
9.0	1.111 ± 0.002	1.103 ± 0.002	−0.72%	1.085 ± 0.002	1.076 ± 0.002	−0.77%
10.0	1.126 ± 0.002	1.116 ± 0.002	−0.92%	1.095 ± 0.002	1.085 ± 0.002	−0.89%

### 3.6 Clinical deliveries

The average gamma analysis passing rates pre‐upgrade were 89.0 ± 5.4% for 2%/1 mm, 99.7 ± 0.3% for 2%/2 mm, and 99.9 ± 0.2% for 3%/2 mm (*k* = 1). Post‐upgrade these values increased to 98.2 ± 1.6%, 100.0 ± 0.0%, and 100.0 ± 0.0%, respectively. The gamma analysis passing rates showed consistency at a criterion of 2%/2 and 3%/2 mm. Passing rates for gamma criteria of 2%/1 mm show a large difference pre‐ and post‐upgrade, with passing rates post‐upgrade being 9.2% higher on average. The distribution of failing points was similar before and after.

## 4 DISCUSSION

### 4.1 Array calibration

#### 4.1.1 Reference calibration

For the daily cross‐calibration field (100.0 cm surface‐axis distance, 100 MU, 4.0 × 4.0 cm^2^), the expected value in the rotational phantom with the SRS top for the X6 CFF beam is 0.920 and 0.950 Gy for the X6 FFF beam. These values remain unchanged from the original commissioning as the pre‐ and post‐upgrade measurements are congruent within the measurement uncertainty. These values can be used for the daily cross calibration.

The array continues to measure absorbed dose accurately under reference conditions, but under non‐reference conditions and VMAT delivery techniques the dose rate can vary and the non‐linear dose rate response, shown in Figure 3, has an impact on the cross calibration of the array. This has been discussed previously for the 1000^SRS^ array^19^ and is an expected behavior of a liquid filled ionization chambers, most likely due to a larger proportion of recombination in measurements at the higher dose rates. This non‐linear dose rate response initially required multiple calibrations for X6 FFF beam, with the appropriate calibration file being selected prior to PSQA based on the average dose rate in the plan.^9^ After the hardware upgrade, only a single calibration file was required for the X6 FFF beam as the dose‐rate dependence reduced from –2.52% to –1.02% (response at 1300 MU/min relative to 100 MU/min). As the majority of clinical plans utilizing X6 FFF will have an overall averaged dose rate of 700 to 1300 MU/min, and the array exhibits a 0.79% variation in response across that range, a 1000 MU/min calibration file provides a balanced compromise of < ± 0.4% uncertainty across that range. Dose non‐linearity is expected to increase for dose rates > 1300 MU/min, as a result additional calibration files may be required for photon beams capable of delivering dose rates > 1300 MU/min.

#### 4.1.2 Relative calibration

The manufacturer's specification for the relative calibration of the array is ± 1.0% and ± 0.5% from the central chamber of the array pre‐ and post‐upgrade respectively. These values were hence chosen for the action and suspension levels in our testing. From these results it can be determined that the majority of the chambers in the 1000^SRS^ array are compliant post‐upgrade, with 100 chambers exceeding the action level for X6 FFF, and 48 for X6 CFF, and only a single off‐axis chamber exceeding suspension level.

This is a large improvement over the pre‐upgrade relative calibration, and the single detector out of 977 exceeding the suspension level is not expected have a clinical effect as the chamber is positioned at the very periphery of the array (Figure 1b). From Figure 1 and Figure 2 it is also clear that the relative calibration of the 1000^SRS^ array is dose rate dependent being better in the X6 FFF reduced dose rate measurements.

### 4.2 Water equivalent depth

Our measurements do not indicate a change to the water equivalent depth of the EPOM pre‐ and post‐upgrade, and it remains within manufacturer's specification of 10.1 mm. Notably though our measurements are incongruent with Poppe et al.^7^ who presented measured values as shallow as 9.5 ± 0.2 mm and states the area density of the inherent build‐up is 1.31 g/cm^2^ (indicating a theoretical water equivalence of 11.8 mm). As the EPOM of a plane‐parallel detector is generally taken to be at the center of the front surface of the sensitive volume,^20^, ^21^ and the thickness of the upper copper electrode and specific composition of the ‘glass reinforced plastic’ build‐up material are not publicly available, it is difficult resolve these differences more conclusively.

### 4.3 Signal leakage

Overall the array shows an improvement in signal leakage post‐upgrade, though leakage is still worse while rotating compared to being stationary. Notably the manufacturer's specifications (2.0 mGy/min) were set as the action level and Table 2 shows a large number of peripheral chambers (up to 17% pre‐upgrade or 10% post‐upgrade) exceeding this level, indicating leakage may still be a weakness of the array's performance. Nevertheless, the array is deemed acceptable for clinical use post‐upgrade as all chambers are within the 10 mGy/min suspension level derived from IPEM guidance.^16^ The cable connecting the array to the DI4000 carries an analogue signal so care must be taken when measuring VMAT deliveries to ensure that it is securely attached and there is enough slack for the array to freely rotate ± 360° without strain.

### 4.4 Dose rate linearity

Results show that the dose rate linearity for the array is outside of manufacturer's specification of ≤ 0.50%. When measured with a Farmer‐type ionization chamber, the dose rate linearity measured was ≤ 0.20% for all beams. As illustrated in Figure 3 the 1000^SRS^ array under‐responds in higher dose rates, though it has noticeably improved after the hardware upgrade.

The DPP at maximum dose rate was 0.619 mGy pre‐upgrade (1270 MU/min), and 0.652 mGy post‐upgrade (1320 MU/min). Although difficult to compare directly; our pre‐upgrade normalized reading of 0.975 is comparable to the result of 0.979 from Poppe et al.^8^ (–2.5% versus –2.1% at DPP of 0.619 mGy). However, our post‐upgrade reading indicates an improvement with a normalized value of 0.990 compared to 0.978 from Poppe et al.^8^ (–1.0% versus –2.2% at DPP of 0.652 mGy).

### 4.5 Output factors

The output factors for the 1000^SRS^ array exhibited a maximum of difference of –0.92% for X6 CFF and –1.2% for X6 FFF when comparing before and after. When comparing to ion chamber measurements, the 1000^SRS^ array appears to diverge at field sizes ≤ 2.0 × 2.0 cm^2^ though consideration must be given to the positional uncertainties at such small field sizes and the different dimensions of the detectors’ active volumes. Furthermore, Lechner et al.^22^ reported residual under‐response from ion chambers in small fields even after volume averaging had been corrected because of material density differences between the active volume, electrode, and surrounding water. The output factors in the SRS top are more clinically relevant than those in solid water, and Figure 4 shows that the 1000^SRS^ array matches the TPS more closely than the ion chamber measurements for fields < 4.0 × 4.0 cm^2^. After the upgrade the array agreed within ± 0.95 % of the TPS from field sizes of 1.5 × 1.5 cm^2^ to 10.0 × 10.0 cm^2^ for both X6 CFF and X6 FFF beam energies.

For field sizes ≤ 1.0 × 1.0 cm^2^ differences between the 1000^SRS^ array and the TPS were > 2.0 % for X6 CFF and X6 FFF beams, which is similar to what Markovic et al.^23^ reported. This was due partly to the previously discussed dose rate dependence of the array. In the 1.0 × 1.0 cm^2^ field the dose rate drops off by approximately 25%–30% compared to the 4.0 × 4.0 cm^2^ field. This reduction in dose rate along with potential volume averaging effects and other small field effects not accounted for in the 1000^SRS^ array measurements result in these errors in the 1.0 × 1.0 cm^2^ field and below. A difference of 2.92% for 1.0 × 1.0 cm^2^ with 5.0 cm build‐up is however comparable to the difference of 2.65% reported by Shin et al.,^10^ though for field sizes ≥ 1.5 × 1.5 cm^2^ our post‐upgrade measurements have decidedly smaller differences than those reported by Shin et al.^10^ As a result, field size dependent correction factors as recommended by Shin et al. are not required and the clinical workflow at our institution is substantially more streamlined.

In conclusion the 1000^SRS^ array is deemed acceptable for use for plan verification where the equivalent field size is greater than 1.5 × 1.5 cm^2^ as it exhibits good agreement (< 0.95%) with the TPS for both X6 CFF and X6 FFF beams.

### 4.6 Clinical deliveries

The gamma analysis passing rates show that the array is capable of proficiently measuring the delivered plan down to a gamma criterion of 2%/2 mm. Results indicate no change to the passing rates for gamma criteria ≥ 2%/2 mm, where the differences pre‐ and post‐upgrade are within uncertainty.

It can be difficult to verify a 1 mm DTA across an extended timeframe as the daily quality assurance action level for our isocentre is 1 mm on linacs used for stereotactic treatments, in addition to geometric uncertainties from the initial measurement set‐up and motion related uncertainty from the phantom rotating during VMAT delivery. A combination of signal leakage (particularly in the low dose or out‐of‐field regions), varying dose rates during VMAT delivery, daily output variations in the linac, statistical noise in the calculated dose cube produced by Monaco, and approximations used to reconstruct the measurement dose cube in VeriSoft can also make verifying a dose‐difference < 2% unreliable with this array.^5^ For plans that require a gamma‐criteria stricter than 2%/2 mm, it is recommended to use an alternative PSQA approach.

## 5 CONCLUSION

The hardware upgrade had a noticeable effect on the response of the array. The signal leakage, relative calibration of off‐axis chambers, dose rate dependence, and output factors have all improved post‐upgrade enabling a streamlined procedure in clinical practice through elimination of multiple calibration files. The changes are overall a great improvement, however the determination of absorbed dose in reference conditions in the central chamber is unaffected. This study was a single center evaluation and expanding the number of plans measured to include multi‐lesion cases and other treatment sites would be beneficial. Notably some dose rate non‐linearity in the array remains and the single calibration file is intended for PSQA with dose rates of 700–1300 MU/min, with increased uncertainty for CFF beams <700 MU/min or FFF beams >1300 MU/min.

## AUTHOR CONTRIBUTIONS

**Morgan Healy**: Writing—original draft; writing—review & editing; conceptualization; methodology; investigation; formal analysis; visualization; validation; data curation. **Michael Roche**: Writing—review & editing; conceptualization; methodology; investigation; formal analysis; validation; supervision; resources; project administration. **Darren Coen**: Writing—review & editing; validation; resources.

## CONFLICT OF INTEREST STATEMENT

The authors declare no conflicts of interest.
